# Well-Being at Work: A Cross-Sectional Study on the Portuguese Nutritionists

**DOI:** 10.3390/ijerph18157839

**Published:** 2021-07-23

**Authors:** Rita de Cássia Akutsu, Ada Rocha, Victor Viana, Luiz Akutsu, Izabel Cristina Silva, Raquel Braz Assunção Botelho, Heesup Han, António Raposo, Antonio Ariza-Montes, Luis Araya-Castillo, Renata Puppin Zandonadi

**Affiliations:** 1 Department of Nutrition, Faculty of Health Sciences, University of Brasília, Brasília 70910-900, Brazil; rita.akutsu@gmail.com (R.d.C.A.); raquelbotelho@unb.br (R.B.A.B.); renatapz@unb.br (R.P.Z.); 2 GreenUPorto, Faculty of Nutrition and Food Sciences, University of Porto, 4150-180 Porto, Portugal; adarocha@fcna.up.pt (A.R.); victorviana@fcna.up.pt (V.V.); 3 Court Union Accounts, Brasília 70042-900, Brazil; luizakutsu@gmail.com; 4 Faculty of Ceilândia, University of Brasília, Brasília 72220-275, Brazil; belbiomedica@gmail.com; 5 College of Hospitality and Tourism Management, Sejong University, 98 Gunja-Dong, Gwanjin-Gu, Seoul 143-747, Korea; 6 CBIOS (Research Center for Biosciences and Health Technologies), Universidade Lusófona de Humanidades e Tecnologias, Campo Grande 376, 1749-024 Lisboa, Portugal; 7 Social Matters Research Group, Universidad Loyola Andalucía, C/Escritor Castilla Aguayo, 4, 14004 Córdoba, Spain; ariza@uloyola.es; 8 Faculty of Business Administration, Universidad Autónoma de Chile, Santiago 7500912, Chile; 9 Facultad de Economía y Negocios, Universidad Andrés Bello, Santiago 7591538, Chile; luis.araya@unab.cl

**Keywords:** well-being at work, nutritionists, Portuguese nutritionists, demographic profile

## Abstract

This exploratory, nationwide cross-sectional study was performed to investigate the well-being of Portuguese nutritionists, in addition to outlining their professional and demographic profile. Descriptive analyses were carried out to determine the measures relating to centralising tendency and dispersion of the sample. We compared means and proportions through *t*-tests and Analysis of Variance (ANOVA). The sample size was 206 individuals, respecting a minimum of eight respondents per item to validate the instrument. We recruited Nutritionists from Portugal nationwide using the list of electronic mail provided by the Order of Nutritionists. We sent an electronic mail to all the Nutritionists registered in this Order. We also used messaging applications and social networks (Instagram, Facebook) to reach Nutritionists who were not accessing electronic mail. Most respondents are women (92.5%), young (mean age = 31.4 ± 8.07 years; 54.2% of participants aging under 30 years), single, and with no children. More than half are Catholic (73.8%) and have less than ten years of nutritionist undergraduate completion (55.4%). The only variable that influences well-being at work is the economic variable Household Monthly Income. Those who earn less than €500.00 per month perceive themselves at a lesser state of work well-being than those who earn from €2501.00 to €5000.00 per month.

## 1. Introduction

Social and economic arguments state that good work is the way to improve the individuals’ well-being and prolonged lack of well-being at work can harm physical and mental health [1]. Once work plays a central role in individuals’ lives, it brings paradoxical consequences to workers’ social, physical, and psychological integrity [2,3,4]. Low job satisfaction is the main cause of turnover among health care professionals [5,6]. Well-being at work is associated with reduced levels of stress, work-related and psychological diseases, burnout, and unhealthy practices (smoking, drinking, overeating, and lack of exercise), and consequently lower levels of non-communicable chronic diseases (NCD) [7]. The idea of subjective well-being comes from the hedonistic tradition and involves a more frequent occurrence of positive than negative effects (emotions and moods) and satisfaction with life. Psychological well-being results from the experience of self-realization (the eudemonic tradition) [8].

In this sense, some researchers are increasingly concerned about studying a working system compatible with human dignity and surrounded by the commitment to human organizations’ well-being [9,10]. Several areas approach such concepts and also reveal different interests when dealing with the subject. For example, health sciences emphasize physical well-being, its signs, and symptoms. Economists are concerned with the well-being of the population through the supply of goods and services, and Psychology focuses on the satisfaction of individual aspirations and expectations to ensure well-being, including at work [8]. Based on the subjective and psychological welfare approaches, some authors have created models that allow welfare analysis from the perspective of Social and Work Psychology [8,9,10,11,12,13]. These authors believe that evaluating well-being contexts, such as work, is more advantageous than evaluating well-being in general because the work context’s background relationships with well-being are strong, contributing to a better understanding and interventions regarding this variable.

The growing interest in well-being in the workplace suggests a possible paradigm shift in the way society thinks about and treats workers’ health. This interest is driven, in part, by studies showing that well-being can lead to more engaged and productive workers [14,15,16].

Waddell and Burton [17] stated, in a review of other studies, that work is generally good for physical health and mental well-being. Unemployment is associated with the opposite and work can reverse the adverse health effects of unemployment. However, many workers aspire to more than just acceptable conditions; well-being can mean more than productivity, job functioning or job satisfaction [18]. By most definitions, well-being goes beyond mere ambition and includes optimism, a life with meaning, prosperity and fulfilling and positive success [19].

Worldwide, healthcare professionals have markedly lower well-being at work than other work sectors, with burgeoning chronic disease burdens [20]. In some countries, there is an increasing interest in improving healthcare professionals’ mental health and well-being, such as that of nutritionists [9,10,21,22,23,24]. Therefore, it is important to know the health professionals’ profile and their status regarding well-being at work in order to improve their health and well-being. Job satisfaction among nutritionists has been studied, but most of these studies focused on nutritionists working in the United States, Canada, South Africa, Sudan, Brazil, and Iran [5,8,25,26,27,28,29,30,31,32,33]. Since well-being at work impacts on work performance and quality of life, knowledge about the profile of well-being at work is essential to improve the working environment and reduce NCD and turnover of nutritionists [5]. Work-related well-being can affect individuals differently, and it is essential to consider socioeconomic and demographic factors approaching when stress-related issues [34,35,36]. Structural inequalities and differences in socio-demographic issues may also affect well-being at work [34,35,36].

To our knowledge, no studies have investigated whether well-being at work is modified by socioeconomic, demographic and educational variables among Portuguese nutritionists, nor the professional path of Portuguese nutritionists, their perception of their work, the educational framework, or their well-being. Since we hypothesize that socioeconomic and demographic variables can influence the well-being of Portuguese nutritionists, this study addressed the research questions: (1) who are Portuguese nutritionists?; (2) what is the perception of well-being at work for Portuguese Nutritionists?; (3) is there a statistical relationship between well-being at work and the socioeconomic or demographic data on Portuguese nutritionists? In this sense, this research aimed to verify Portuguese nutritionists’ professional and demographic profile and the influence of socioeconomic and demographic characteristics on well-being at work. This work was based on Paz et al. [13] and Akutsu and Paz [8], who conceive well-being as a process, defining it as the satisfaction of needs and individuals’ fulfillment of desires as these play a role in their profession. This concept takes into account the role of work organizations in individuals’ health and in developing healthy environments that enable positive relationships and attitudes. It is expected that understanding of the factors that influence nutritionists‘ well-being may lead to greater professional valorization. Potentially, knowledge of professional well-being among nutritionists may lead to effective avenues to prevent or manage nutrition-related chronic diseases, and improve the public’s trust in nutritionists and the interprofessional healthcare team’s dynamics.

## 2. Materials and Methods

This cross-sectional research was performed using a previously validated instrument [8] to investigate the well-being of Portuguese nutritionists, in addition to outlining their professional and demographic profile. The Research Ethics Committee of the University of Porto approved this study through Registration No. 28/CEUP/2016.

For this study, the instrument which initially presented a questionnaire composed of 14 items (Cronbach’s alpha of 0.91) was expanded to adapt to Portuguese Nutritionists using a theoretical review that pointed to the addition of 12 items (constructs) based on well-being precedents. For the present study, the original instrument [8] was submitted to reverse translation for emic/etic adjustment [37]. After this stage, the instrument was analyzed by eight Portuguese nutritionists, selected by convenience, for confirmation, removal, or inclusion of items. Therefore, the instrument underwent validation by the Delphi Technique with Focus Group (composed of six Portuguese nutritionists and two psychologists) in two rounds [38]. Demographic variables were included according to the study of Akutsu et al. [8] and after a literature review of the antecedent studies of well-being. They were also submitted to the nutritionists participating in the focus group to verify the variables’ relevance. In particular, the religion variable was included after considering studies that contemplated this variable, and was widely discussed by focus group participants [39,40,41]. There is evidence for religiosity/spirituality being positively related to various mental health indicators, including subjective well-being and personality dimensions [40]. Also, religiosity/spirituality is considered important in health and well-being and the process of dealing psychologically with an illness [40].

All items added to the instrument were maintained after performing the reliability and relevance analysis. The instrument adopted a five-point Likert scale ranging from Never (0) to Rarely (1), Sometimes (2), Often (3), and Always (4).

### 2.1. Sample

After the Delphi Technique’s validation with the Focus Group, we recruited Nutritionists from Portugal nationwide using the list of electronic mail provided by the Order of Nutritionists. It is important to highlight that we sent the electronic mail to all registered Nutritionists (n = 2800). Besides, we used messaging applications, and social networks (Instagram, Facebook) to contact Nutritionists who were not accessing electronic mail. In this sense, we invited health professionals, researchers, and influencers to publicize the study. Volunteers received, along with the research link, an invitation to participate, as well as the Consent Form. To be a nutritionist and to work in a specific area of nutrition were the inclusion criteria. The questionnaire was sent to them through an email link in order to access the Survey Monkey^®^ platform, where they signed a Term of Free and Informed Consent and completed the research instrument.

For validation and statistical analysis of the questionnaire for Portuguese Nutritionists, the study of Hair et al. [42] was considered, which suggests 200 professionals per instrument, and Pasquali’s study [37], which suggests eight respondents per instrument item. Therefore, we conducted the study with a total of 206 individuals with a confidence interval of 95%, and an error of 7%. Moreover, we also used the items to investigate socioeconomic and demographic characteristics from the original study [8] for the characterization and differentiation of the sample (gender, age, marital status, religion, income, and academic background).

### 2.2. Data Treatment and Analysis

We conducted an exploratory factorial analysis using the Principal Axis Factoring model (PAF) with PROMAX rotation to verify the instrument’s psychometric quality. In the correlation matrix, in addition to Bartlett’s test of sphericity, we calculated the Kaiser-Meyer-Olkin (KMO) value, which indicates the suitability of the sample. KMO values above 0.871, 0.881, 0.881 and 0.884 are considered good, great, and excellent, respectively [43]. The factorial loads’ size expressed the variables’ validity, and a minimum factor load of 0.30 was determined to accept the item [43]. 

Cronbach’s alpha test was performed to analyze the scales’ reliability or internal consistency [43]. Once the factors of the scales were confirmed, the mean scores were calculated for each respondent.

We used descriptive analyses to determine the measures of central tendency and dispersion of the sample. We compared the sample’s means and proportions through *t*-tests, Chi-squared test, and Analysis of Variance (ANOVA).

## 3. Results

The final instrument presented an alpha value of 0.92, which is considered an excellent psychometric index [44] with 26 items and four factors. The questions were distributed into four factors. The first one is related to exterior perception with questions 2, 4, 6, 7, 11, 15 and 26. The second factor is about the nutritionists’ perception in itself (questions 1, 3, 5, 8, 13, 14, 18 and 21). The third factor is about task perception (questions 19,20, 22, 23 and 24). Finally, the fourth factor concerns perception related to the nutritionists’ category (questions 9, 10, 16 and 17). The final sample of this study includes 206 individuals—7.4% of the registered nutritionists in Portugal—(mean age = 31.4 ± 8.07 yeas), which is considered adequate by Hair et al. [42] (who suggest 200 professionals) and Pasquali [43] (who suggests eight respondents per construct).

The normality premises of all variables were also verified, and the results pointed to normal behavior. For this reason, we performed comparisons of means and variances (ANOVA) of Wellbeing at Work with all socioeconomic and demographic variables. 

Portuguese nutritionists often perceive themselves in a situation of workplace well-being. The mean and the standard deviation of Well-being at Work of Portuguese Nutritionists measured by central tendency and variance were 2.59 ± 0.47. Table 1 shows the sociodemographic characteristics of Portuguese nutritionists. Most respondents are women from 25 to 29 years old, with a higher education degree, single, and without children (Table 1).

Most of the respondents (67.6%) are currently working in the clinical area and work in more than one workplace (61.2%). Most of the respondents finished their undergraduate degree at Public Universities, and they graduated in less than ten years (55.4%). Most of the nutritionists work in private companies/professional practice (58.9%); however, the workplace did not influence well-being at work and the variables mentioned earlier. The only variable that influenced Well-being at Work was the economic variable, Household Monthly Income (Table 2) by Variance Analysis and Student’s *t*-test. Those who earn less than €500.00 per month perceive themselves at a lesser state of work well-being than those who earn from €2501.00 to €5000.00 per month.

Analyzes were performed to verify the relationship between family income and gender, and area of practice and age. The family income range does not change by gender (*p* = 0.313), but the area of practice and age influences it. Older professionals (*p* = 0.001) who work in the teaching and clinical areas (*p* = 0.001) have a higher family income.

Well-being at work for Portuguese nutritionists was positive, above 2.5, the mean point of the scale. By analyzing by factor, factor 1, related to exterior perception, had the lowest mean and was below the mean point (2.09 ± 1.07), indicating that the social perception of work influences well-being at work. Factor 2 presented a mean score of 2.77 ± 1.05, and factor 4 2.69 ± 0.99. The highest mean value was 2.96 ± 0.84 for factor 3 (task perception). 

Income was the only variable that showed different well-being at work scores. This tendency was also observed for factor 1 scores. Nutritionists with monthly incomes lower than €500.00 presented a mean score of 1.62 ± 1.11 for factor 1, statistically different from all other household family income scores. Nutritionists with a monthly income between €500.00 and €1000.00 presented a mean factor 1 score of 2.08 ± 1.07; between 1001.00 and 2500.00 a mean score of 2.06 ± 1.16; and from 2500.00 and 5000.00 a mean score of 2.40 ± 1.07. This tendency was not observed for factor 3, which presented the highest mean score for the whole sample. When comparing nutritionists by income, no statistical differences were observed for factor 3 scores.

## 4. Discussion

This study first described Portuguese nutritionists’ characterization and professional path, their perception of their work, the educational framework, and their well-being at work. This is important due to the increasing number of nutritionists in Portugal [45,46]. At the moment of data collection, there were 2800 nutritionists registered in Portugal. From them, a convenience sample of 206 (7.36%) nutritionists participated in our study. Most of the respondents finished their undergraduate degree less than ten years ago, probably due to the recent implementation of the undergraduate Nutritionist course in private Universities. This first appeared in Portugal in 1975 at the University of Porto. In 1983, it evolved to a licentiate degree, and the first students were granted their degrees in Nutrition Sciences in 1988. Until 2004, the course existed only at the University of Porto, then the degree was created in other private universities [47,48,49]. It is important to emphasize that developing a professional identity occurs through a professionalization process, and formal academic education and a subsequent apprenticeship or training are considered important tools for professionalization [50]. However, a review study conducted by MacLellan et al. shows that little research has been conducted in the process of professionalization in dietetics [50]. In our study, only 14.8% of nutritionists work in the public health area. Most of the respondents (67.6%) are currently working in the clinical area, as shown by other studies [5,6,30] and they work in more than one workplace (61.2%) [51].

Besides female hegemony (92.5%) within the nutritionist profession in Portugal (Table 1), our study shows that, in this country, nutritionists are young, most of whom (54.2%, n = 140) are below 35 years old. Studies have demonstrated that female hegemony among nutritionists is common in different countries [5,6,10,30,49,52], including Portugal [53]. The combination of gender and age in association with low qualification probably contributed to the low wages earned by Portuguese nutritionists and those in other countries [54,55]. The wage level is far from that earned by American female nutritionists, whose individual wage averages €3150.00 per month [56]. In Portugal, nutritionists earn a slightly lower wage than Brazilian nutritionists, probably due to the Brazilian nutritionists’ higher degree of specialization. In Brazil, 73% of nutritionists are postgraduate [8,51], higher than we found among Portuguese nutritionists (Table 1). Our study showed that younger nutritionists (most of our respondents) receive lower wages because they are less skilled and less advanced in their careers. Therefore, they accept positions that pay less than those who have worked as nutritionists for more time. In this sense, the lower wage among nutritionists can be justified by their basic business skills, while medical nutrition skills have expanded. Therefore, most nutrition practitioners serve in staff rather than leadership/management positions resulting in leadership roles in food and nutrition services being filled by other professionals [57]. A study conducted in the USA with 521 nutritionists showed that they perceive that they lack power in the workplace, in which physicians held the most power [58]. Lack of power is considered an important limiting factor in achieving overall professional potential [59]. Nutritionists need to understand the concept and the use of power in the workplace. Basic skills and concepts related to power must be incorporated into nutrition continuing education using a gender-focused teaching approach [58]. Therefore, a continuous training program should be applied to increase the nutritionists’ knowledge and skills, motivating them by promotion and a higher salary level [5]. 

Female hegemony in the profession has repercussions on career, social prestige, and income [51]. Mayer et al. [60] stated in their study that nutritionists were mostly female and that although the labor market has been increasing, the new jobs were frequently (86%) part-time. not only because females need to conciliate career and family care, but also because these positions receive lower payment. 

The other socioeconomic and demographic analyzed variables within the context of Personal Well-being at Work showed that most, as young professionals, are not married, do not have children, and usually live with two other people, which reinforces the change of paradigm concerning the professionalization of women in general, and nutritionists in particular [52]. Such change is observed in societies because of the change in women’s role at work [61] and, above all, the change in the meaning of work itself [62].

The growth of obesity among adults and children has been reported worldwide [63,64,65] and has affected nutritionists’ careers due to the growing demand for professionals whose expertise is in the field of nutrition, despite efforts made by the nutrition sciences and nutritionists to reduce obesity. Nutritionists have expanded their field of work by seeking to occupy political spaces in the development of public policies that propose advances in overweight reduction and, moreover, policies that focus on the right to adequate and healthy nutrition [65,66]. The strategies adopted by governments have been influenced by nutritionists in several instances [67,68,69]. If, on the one hand, they have affected labor growth, they also affect labor relations and these professionals’ well-being.

Studies on well-being at work (7.59) point to the importance of this construct and show strong evidence of a positive association between good health (including healthy behaviors) and well-being. Portuguese nutritionists often see themselves in a situation of personal well-being at work, and they revealed that the most central aspects of such a perception are the importance of work for themselves, their clients/users/students, and society. Some studies showed that while nutritionists experience low levels of desensitization to human needs and moderate emotional exhaustion levels, they also experience only a moderate sense of personal accomplishment (which measures their sense of achievement on the job and quantifies their perception of influencing others) [6,70,71].

The appreciation of these aspects and, consequently, their positive influence on personal well-being at work was expected, since nutritionists, as health practitioners, have a hierarchy of work values characterized as altruistic characteristics [8]. Studies also show that men and women have different expectations about work, and this is due to different motivations and the appreciation they give to work, which can lead to greater or lesser well-being and job satisfaction, staff turnover, and professional performance [72,73]. These gender differences certainly affected the perception of Portuguese nutritionists of personal well-being at work because we cannot analyze nutritionists’ professional practice in Portugal or worldwide without considering that women correspond to more than 90% of nutritionists. Some authors stated that the association of low wages to the gender issue could not be ignored, while several studies point to it [74,75]. However, in our study, family income does not change by gender (*p* = 0.313).

In this sense, the constructs that obtained the worst means were the perception of justice regarding wages, the perspective of change, and skills reward. Similar data were found by Akutsu and Paz [8] in their research with a sample of 560 Brazilian nutritionists and Ferreira et al. [10] with a sample of 249 nutritionists from São Paulo, Brazil. These studies reinforce how connected are the issues of gender, wage-earning, and personal well-being at work.

The study by Gaião [75], conducted with 118 Portuguese nutritionists, also indicates low wages earned by professionals, similar to our study, which has been repeatedly pointed out [76,77] as a factor of non-satisfaction at work, as well as the studies of Akutsu and Paz [8] and Ferreira et al. [10], previously mentioned, of malaise at work. Therefore, improving nutritionists’ well-being at work, better payment, and other types of workers’ compensation seem to be necessary, particularly those related to rewarding skills [5,31].

Besides paying fair remuneration and promoting workers’ stability, employers should help nutritionists achieve good health conditions by encouraging physical activity, supporting healthy eating habits, and facilitating the balance between work, social life, and family [74].

Nutritionists, like other workers, have experienced an era of changes at work [78]. Among those changes, we can mention the need to refresh technical knowledge in the nutritionists’ area of practice and the incorporation of new technologies [79]. Moreover, it is necessary to consider work instability and the strong influence of gender in labor activities and women’s wages in Portugal and worldwide [11,80].

Due to the multidimensionality of the Personal Well-being at Work construct, identity resembles a process of identification in an interplay between the individual’s self-concept and one’s social representations, i.e., the individual’s perception of self insertion in a given society, and what that society assigns as characteristics [11,81].

Considering that work has a dominant role in constructing the individual’s professional identity, this concept can also be defined as the individual’s representation and what others accredit to such person regarding, the work this person performs [81]. That is why professionals and technical nutrition associations must join forces to fight for equal pay, fair working conditions, and proper training.

Professionalization is an achievement of a particular social group and, as such, it creates an occupation with prestige and special power that holds cognitive and normative authority through political, cultural, and ideological means [82]. To become a professional, many allied health occupations have focused upon acquiring the traditional attributes of a profession, neglecting professionalization. Therefore, individuals of allied health occupations that claim professional status may experience the problems of lack of occupational prestige, limited opportunity for vertical mobility, and occupational burnout [71]. While cognitive authority is due to education, specific knowledge, proper language, and the effective capacity to solve society’s problems, normative authority is due to self-discipline skills and the ability to fulfill the regulation of conduct spontaneously [76]. Once acquired, such characteristics guarantee autonomy and, consequently, the recognition of professional identity by society.

Therefore, the professional identity of nutritionists is established in the process of continuous construction based on the fulfillment of the role for which one was initially prepared and, over time, through several transformations by developing areas of competence, goals, and values that identify a person with particular professional inclinations [83].

In this way, it is essential to have a sense of belonging to a professional group that holds the necessary knowledge, language, mission, and, of course, the tools required for professional development in order to be recognized by the society in which it belongs [83].

Our study’s strength is that we recruited individuals from the entire country to participate, attracting individuals from the Portuguese Nutritionists nationwide, which enabled us to generate more consistent data in this area. However, this study presents some limitations since we used a convenience sample. Therefore, the fact that subjects were not randomly selected presents a potential bearing on whether those answers through email and social networks are representative of the Portuguese nutritionists as a whole, even though we sent the questionnaire to emails of all registered Portuguese nutritionists. Moreover, random sampling makes it difficult to attract enough participants. Participants were self-selected, and perhaps those with a low degree of well-being at work were too overwhelmed or busy to participate in the research. The sample for this study consisted of practicing nutritionists. If nutritionists had left the profession due to low well-being at work, they would not have been included.

The fact that our sample had a higher proportion of female respondents could be another limitation. Other studies with Nutritionists also showed this trend since graduates consist mostly of women [5,10,30,49,52]. Women also tend to participate more in surveys than men [84,85]. Besides, due to the study’s cross-sectional nature, causal relationships cannot be established, limiting the discussion. Further study is necessary to assess the relationship between well-being at work and the need for intervention. Another potential limitation of our study is that employees are not randomly assigned to workplaces. Failure to account for sorting of employees may bias estimated effects for the measures of well-being at work. This problem can be addressed in future studies, including information on employees’ wages and work histories.

Self-reports are researchers’ favorite data collection method due to their low cost, easy use and flexibility, and they enable researchers to verify behaviors that could not otherwise be observable [86,87]. However, self-report measures may be prone to exaggeration since individuals tend to overreport their information, mainly because of the social desirability bias, a critical limitation of self-report measures [86,87]. Therefore, construct validity is essential with the use of self-reports. Construct validity refers to the degree to which a measure accurately assesses the intended construct. The establishment of construct validity is an essential requirement for any rigorous line of research [86], as performed in this study, to reduce bias.

## 5. Conclusions

This cross-sectional study is the first that analyzed Portuguese nutritionists’ professional and demographic profile and their well-being at work. Most Portuguese nutritionists are young women, single, and with no children. The hypothesis that socioeconomic profile influences the well-being of Portuguese nutritionists was confirmed by the interference of income on well-being. The only variable that influenced well-being at Work was the economic one (Household Monthly Income). Therefore, to improve Portuguese nutritionists’ well-being at work, better payment and other types of workers’ compensation seem to be necessary because low levels of well-being at work might result in poor mental and physical health and decrease work productivity. 

Probably, income improvement could come through professional qualification since most nutritionists only have an undergraduate degree in nutrition. The Order of Nutritionists is recent in Portugal and needs more time to present a better structure and improve career patterns. In addition, it is necessary to increase the number of professional associations (unions and councils) to fight for better salaries for this professional category. Health policymakers should discuss the role of health professionals as a multidisciplinary team, highlighting the importance of each category to the health system, as nutritionists still feel little recognized for their work.

Future studies are necessary to evaluate the relationship between Portuguese nutritionists’ well-being at work and productivity, mental and physical health. This search may open doors for more studies in the field of well-being regarding nutritionists’ work and the potential impact of nutritionists’ work on psychosocial aspects and quality of life.

## Figures and Tables

**Table 1:** Socioeconomic and demographic variables of Portuguese nutritionists.

Variable		n *	%
Gender (n = 201)	Female	186	92.5
	Male	15	7.5
Age group (n = 199)	21 to 24 y/o	38	18.9
	25 to 29 y/o	71	35.3
	30 to 34 y/o	31	15.4
	35 to 39 y/o	29	14.4
	40 to older	30	15.9
Level of education (highest degree) (n = 200)	Undergraduate	132	66
	Graduate	54	27
	Residency	13	6.5
	Master’s	1	0.5
Marital status (n = 199)	Single	136	68.3
	Married	52	26.2
	Divorced	11	5.5
Children (n = 201)	Yes	60	29.9
	No	141	70.1
Household monthly income (n = 191)	<€500.00	12	6.3
	From €501.00 to €1000.00	50	26.2
	From €1001.00 to €2500.00	102	53.4
	From €2501.00 to €5000.00	27	14.1
Number of household members (n = 194)	1	47	24.2
	2	51	26.3
	3	56	28.9
	4	32	16.5
	>5	8	4.1
Area of Practice (n = 182)	Clinic	123	67.6
	Teaching	13	7.1
	Foodservice Administration	19	10.4
	Public Health	27	14.8
Type of company of your professional practice (n = 197)	Public	76	38.6
	Private	116	58.9
	Mixed Economy	5	2.5
Number of workplaces at the moment (n = 196)	1	76	38.8
	2	49	25
	3	71	36.2
Type of institution where you finished your undergraduate degree (n = 200)	Public	141	70.5
	Private	59	29.5
Year of undergraduate completion (n = 195)	Before 1996	16	8.2
	1996 to 2004	34	17.4
	2005 to 2009	37	19
	2010 to 2014	80	41
	From 2015 on	28	14.4
Have you changed your area of practice after finishing your last degree? (n = 195)	Yes	54	27.7
	No	141	72.3
Did you work before finishing your undergraduate degree? (n = 197)	Yes	33	16.8
	No	164	83.2

* The number of respondents can vary in each item since the responses were not mandatory in order to continue the questionnaire.

**Table 2:** Variance analysis of well-being at work and of socioeconomic and demographic variables of Portuguese nutritionists.

Variable		Well-Being at Work		
		Mean	Standard Deviation	*p*
Gender	Female	2.61	0.47	0.456 ^$^
	Male	2.43	0.47	
Age group	21 to 24 y/o	2.57	0.42	
	25 to 29 y/o	2.58	0.47	0.330 ^#^
	30 to 34 y/o	2.70	0.45	
	35 to 39 y/o	2.58	0.57	
	40 to older	2.52	0.63	
Level of education (highest degree)	Undergraduate	2.58	0.46	0.658 ^#^
	Graduate	2.57	0.47	
	Residency	2.63	0.48	
	Master’s	2.70	0.48	
Marital status	Single	2.58	0.44	0.783 ^#^
	Married	2.63	0.52	
	Divorced	2.85	0.52	
Religion	Catholic	2.63	0.43	
	Protestant	2.89	0.44	0.244 ^#^
	Agnostic	2.45	0.49	
	Others	2.58	0.55	
Children	Yes	2.70	0.54	0.437 ^$^
	No	2,57	0.44	
Household monthly income	<€500.00	2.29a	0.50	
	From €501.00 to €1000.00	2.57	0.37	0.006 *^,#^
	From €1001.00 to €2500.00	2.58	0.47	
	From €2501.00 to €5000.00	2.82b	0.47	
Number of household members	1	2.58	0.47	
	2	2.67	0.42	0.335 ^#^
	3	2.59	0.45	
	4	2.63	0.36	
	>5	2.67	0.92	
Area of Practice	Clinic	2.58	0.45	
	Teaching	2.72	0.45	0.468 ^#^
	Restaurant Administration	2.48	0.67	
	Public Health	2.67	0.42	
Type of company of your professional practice	Public	2.60	0.48	0.403 ^#^
	Private	2.60	0.47	
	Mixed Economy	2.87	0.20	
Number of workplaces	1	2.58	0.49	
	2	2.63	0.40	0.467 ^#^
	3	2.65	0.49	
Institution type where you finished your undergraduate degree	Public	2.58	0.47	0.473 ^$^
	Private	2.69	0.46	
Year of undergraduate completion	Before 1996	2.77	0.36	
	1996 to 2004	2.56	0.57	0.330 ^#^
	2005 to 2009	2.66	0.46	
	2010 to 2014	2.59	0.39	
	From 2015 on	2.60	0.44	
Have you changed your area of practice after finishing your last degree?	Yes	2.70	0.39	0.237 ^$^
	No	2.58	0.50	
Did you already work before finishing your undergraduate degree?	Yes	2.72	0.42	0.321 ^$^
	No	2.58	0.47	

^$^ Student’s *t* test; ^#^ ANOVA one-way; * *p* < 0.05.

## Data Availability

The study did not report any data.
